# The leaves fall, yet the tree endures

**DOI:** 10.1007/s00018-025-05874-8

**Published:** 2025-10-28

**Authors:** Miloslava Fojtová, Petra Procházková Schrumpfová, Jiří Fajkus

**Affiliations:** 1 Mendel Centre for Plant Genomics and Proteomics, CEITEC - Central European Institute of Technology, Masaryk University, Brno, 625 00 Czech Republic; 2 Laboratory of Functional Genomics and Proteomics, National Centre for Biomolecular Research, Faculty of Science, Masaryk University, Brno, 625 00 Czech Republic

**Keywords:** Plant aging, Plant senescence, Meristem, Telomere biology, Epigenetics

## Abstract

Aging in plants presents a paradox: while individual modules such as leaves and reproductive organs undergo senescence, the plant as a whole may display extraordinary longevity, enabled by its modular architecture and perpetually active meristems. This review explores aging and senescence in plants by challenging commonly held assumptions and integrating emerging insights from telomere biology and epigenetic regulation. We critically examine the role of telomere length as a determinant of replicative lifespan, arguing that its importance is often overstated, particularly in the context of plant systems where telomerase activity persists in meristematic tissues. In contrast, the epigenetic landscape—including DNA methylation, histone modifications, chromatin remodeling, and non-coding RNAs—plays a dynamic and increasingly appreciated role in orchestrating senescence at cellular and organ levels. We synthesize current understanding of how these chromatin-level mechanisms interact with developmental cues and environmental stresses to regulate genome stability, transcriptional reprogramming, and longevity. By integrating chromosomal and epigenetic processes, this review provides a refined conceptual framework for understanding plant aging and highlights new opportunities to enhance resilience and lifespan in crops and long-lived species through targeted manipulation of telomere maintenance and epigenetic pathways.

## Introduction

The lifespan of an organism is commonly defined as the period from birth (or inception) to death [1]. While the terms “aging” and “senescence” are often used interchangeably in everyday language, they refer to distinct biological phenomena. **Aging** describes the progressive, time-dependent decline in physiological function and regenerative capacity over an organism’s life, encompassing molecular, cellular, and systemic changes that collectively increase vulnerability to functional impairment and death [2–4]. In contrast, **senescence** denotes specific, genetically programmed processes—at the cellular or organ level—that result in degeneration, irreversible cell-cycle arrest, or programmed cell death. Although cellular senescence is a hallmark of organismal aging in many multicellular organisms, it can also be induced independently of chronological age, in response to developmental cues or environmental stressors [5]. Senescence can be further categorized based on cellular behaviour [6, 7]. **Chronological senescence** refers to the duration that non-dividing or quiescent cells remain viable, typically reflecting the accumulation of oxidative or metabolic damage. **Replicative senescence**, by contrast, describes the finite number of cell divisions a cell can undergo before permanently exiting the cell cycle, a process often linked to telomere shortening and the accumulation of replication-associated DNA damage [7]. However, emerging evidence suggests that the role of telomere attrition in replicative aging may be overstated.

Both organismal aging and cell- or tissue-specific senescence can be assessed quantitatively using various physiological and molecular biomarkers. Across the tree of life, the interplay between aging and senescence is highly variable. For instance, in mammals, cellular senescence involves activation of p53 and p16^INK4a^ pathways, leading to stable cell-cycle arrest [8]. The systemic accumulation of senescent cells contributes to chronic inflammation and tissue dysfunction, accelerating organismal decline.

Although aging and senescence are mechanistically linked, they are distinct concepts, with overlapping but non-identical manifestations that differ across taxa and biological contexts. While numerous publications and thematic collections (e.g. [9]), address aging and senescence in a wide range of organisms, plants are often neglected, with some notable exceptions [10–13].

Yet, plants offer a unique opportunity to re-examine and refine prevailing theories of biological aging. The distinction between aging and senescence is particularly striking in plants. As in other organisms, plants execute tightly regulated senescence programs in specific organs and cell types—such as leaves, petals, and roots—but their meristematic tissues maintain lifelong proliferative capacity, supporting indeterminate growth and continuous organogenesis [14]. Consequently, defining aging in plants is far more challenging. Plant bodies are inherently **modular**, comprising reiterated units such as stems, roots, and leaves. Many species propagate vegetatively, forming expansive clonal colonies in which identifying a single “individual” and measuring its chronological age becomes difficult [11, 12].

**Fig. 1:**
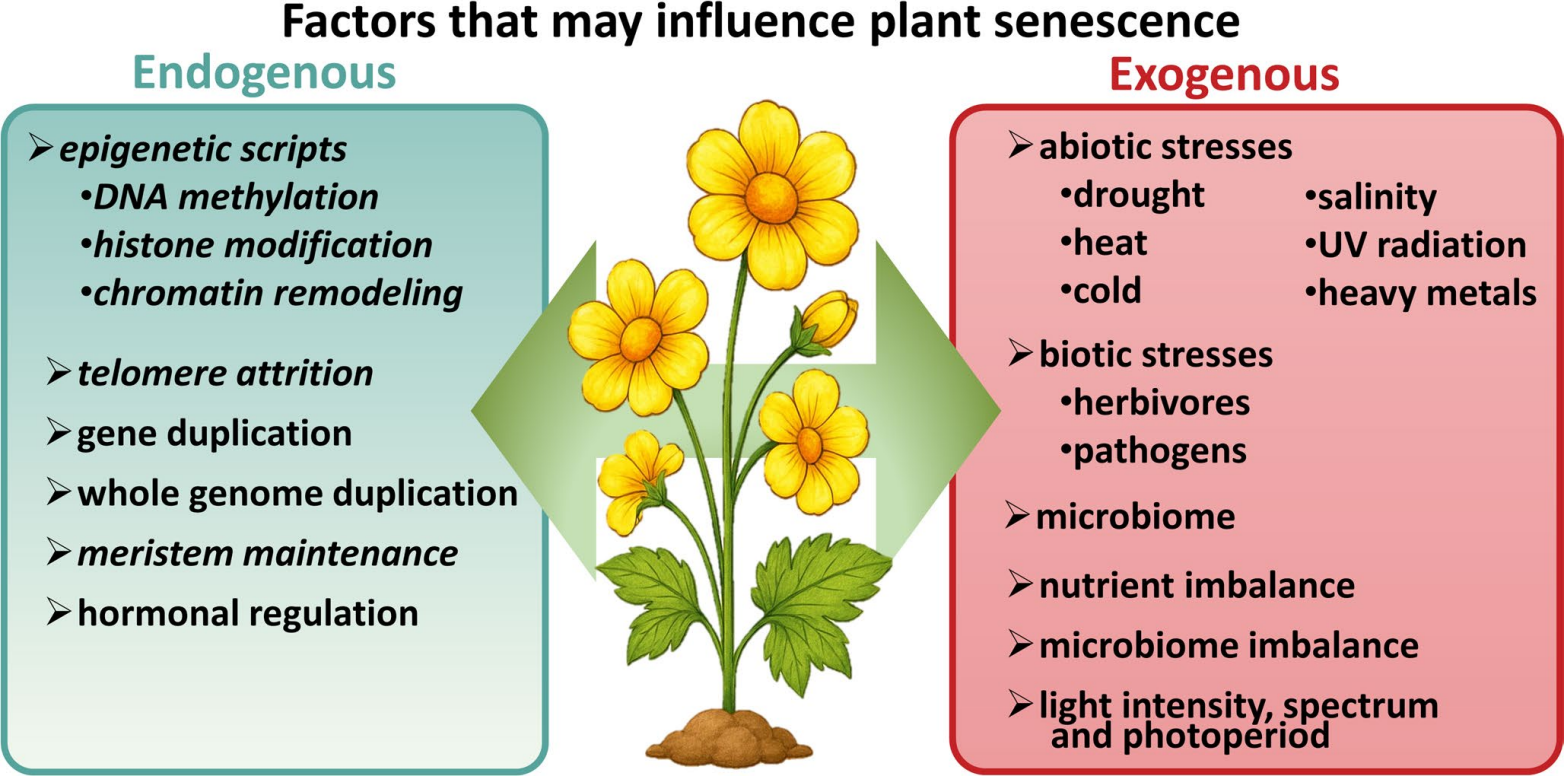
Schematic overview of endogenous and exogenous factors influencing plant aging. The diagram illustrates the major categories of internal (endogenous) and environmental (exogenous) factors that regulate plant aging. Endogenous influences include epigenetic mechanisms, telomere attrition, gene and whole-genome duplication events, meristem maintenance, and hormonal regulation. Exogenous factors encompass a wide range of abiotic and biotic stresses, microbiome dynamics, nutrient imbalance, and light conditions. The factors addressed in this article are indicated in italics

Current knowledge of plant aging and senescence encompasses a diverse set of molecular, cellular, and evolutionary mechanisms, including both longevity determinants and organ- or tissue-specific senescence programs (Fig. 1). As sessile organisms, plants must continuously respond to fluctuating abiotic and biotic stresses—including changes in temperature, light, water availability, pathogens, herbivory, and nutrient status—all of which can influence lifespan [15, 16]. To cope with such pressures, plants have evolved multiple strategies to promote resilience and longevity. These include **gene duplication** and **polyploidy**, often culminating in whole-genome duplications (WGDs), which have introduced extensive redundancy into regulatory gene families across land plant genomes [17, 18]. This redundancy provides a buffer against deleterious mutations, helping to preserve core genetic functions [19]. Remarkably, meristematic cells are further protected from mutational load (as discussed below). Additionally, land plant genomes encode unique metabolic pathways and hormone signaling networks that underpin developmental plasticity and environmental adaptability [20] —traits that may explain why some plant species exhibit unparalleled longevity. Classical reviews on plant aging and senescence have tended to emphasize oxidative stress and redox signaling, environmental cues, hormonal regulation, transcription factor networks, large-scale transcriptional reprogramming, and autophagy-mediated nutrient recycling [21–29]. While these factors are undoubtedly important, this review focuses on two underappreciated or misrepresented mechanisms that operate at the chromosomal and chromatin levels: **telomere maintenance** and **epigenetic regulation**.

Notably, meristematic tissues in plants sustain proliferative potential throughout life, supported by persistent **telomerase** activity. Telomerase has traditionally been associated with telomere length preservation and the delay of replicative aging. However, recent findings suggest that telomere length alone does not fully explain cellular longevity in plants. Instead, dynamic **epigenetic modifications**—including DNA methylation, histone modifications, and chromatin remodeling—appear to play a central role in modulating aging and senescence. By integrating insights into telomere biology and epigenetic dynamics within organ-specific and environmental contexts, this review aims to elucidate general principles governing plant longevity and senescence. We also highlight promising avenues for enhancing lifespan and stress resilience in crops and long-lived plant species through the manipulation of telomere and chromatin-based regulatory pathways.

## Plants hold records in longevity

While certain animal species exhibit remarkable longevity—often associated with low metabolic rates and cold environments—plants frequently surpass animals not only in lifespan but also in the diversity of persistence strategies, including those effective in hot and arid ecosystems. Among animals, *Anoxycalyx joubini* (the Antarctic volcano sponge) may Live for up to 15,000 years [30], and the *Somniosus microcephalus* (Greenland shark) can reach Lifespans nearing 400 years [31]. Mammalian lifespans are generally shorter, with the longest recorded being *Balaena mysticetus* (bowhead whale), estimated at 211 years based on embedded 19th-century harpoon fragments [32, 33].

**In sexually reproducing plants**, which undergo an alternation of generations between diploid sporophyte (2n) and haploid gametophyte (1n) phases, individual life cycles are more clearly delineated. A full generation is typically defined by the sequential completion of both phases. Plant aging can be monitored using biomarkers such as the ratio of photosynthetic to non-photosynthetic tissue, cambial activity, pollen viability, seed yield and mass, germination rates, seedling biomass accumulation, changes in endogenous phytohormone levels, and somatic mutation load [34, 35].

Patterns of senescence and death differ significantly between long-lived **monocarpic** and **polycarpic** species [35]. Polycarpic perennials reproduce multiple times and maintain both indeterminate vegetative apices and determinate reproductive meristems [36]. In contrast, monocarpic species complete only a single reproductive cycle before death, a strategy typical of annuals. However, in annuals, modular units may senesce and be replaced without affecting overall performance or reproductive success [11, 37], —i.e., independently of whole-plant death. Consequently, researchers often focus on **organ-specific senescence markers**—such as upregulation of senescence-associated genes (SAGs), hormonal shifts, and programmed cell death pathways—while conceptualizing “plant aging” as an emergent property of continuous module renewal, rather than a uniform, organism-wide decline [14, 38]. Long-lived monocarpic species are rare; an example is the bamboo *Phyllostachys nigra* var. *henonis*, which undergoes synchronous flowering approximately every 120 years, followed by widespread senescence and death. The next flowering event is expected around 2028 [39].

Among the longest-lived organisms are gymnosperms. *Pinus longaeva* (bristlecone pine) includes individuals aged up to ~ 4,750 years, making it one of the oldest known sexually reproducing plants [34]. Despite localized organ senescence and continuous organ turnover, whole-plant senescence in these trees is negligible, as evidenced by stable biomarkers such as cambial activity, seed viability, and seedling biomass. Other long-lived gymnosperms include *Sequoiadendron giganteum*, *Dacrydium franklinii*, and *Juniperus communis*, with Lifespans ranging from 2,000 to over 3,200 years [12]. Among angiosperms, *Adansonia digitata* (African baobab) is known to surpass 2,000 years in age. However, between 2005 and 2021, a significant mortality event linked to climate change resulted in the death of several of the oldest and largest baobab individuals [40, 41].

**Vegetative reproduction**, a form of asexual propagation, enables plants to persist and spread without completing the full alternation of generations. Clonal offspring are generated from somatic tissues—such as stems, roots, or leaves—producing genetically identical individuals. This strategy ensures clonal fidelity, circumvents reproductive barriers (e.g., triploidy-induced sterility), and enhances survival in environments where seed germination is limited by abiotic stress. Consequently, clonally propagated lineages can persist for tens of thousands of years, blurring the lines of what constitutes an individual organism or its lifespan.

*Lomatia tasmanica* W.M. Curtis, an endangered species with a single known population, reproduces exclusively via vegetative means. Its triploid genome likely accounts for its sterility and genetic uniformity [42]. Fossilized leaves identified as *L. tasmanica* and dated to at least 43,600 years ago suggest that this clonal lineage may be one of the oldest living plant individuals [43]. Similar longevity has been documented in other clonal systems. *Larrea tridentata* (creosote bush) in the Mojave Desert forms a ring-shaped clonal colony estimated to be ~ 11,700 years old, based on growth ring and spatial propagation analyses [44]. In the boreal zone, a *Picea abies* (Norway spruce) clonal population in krummholz form on Fulufjället Mountain, Sweden, has been radiocarbon-dated to ~ 6,500 years, representing the oldest known clonal tree-forming gymnosperm [45].

The clonal colony known as **Pando**, comprising *Populus tremuloides* in Utah, is often cited as one of the oldest Living organisms. Initial age estimates ranged from 80,000 to 1 million years [46], though more recent analyses suggest these may be overestimations [47–49]. Regardless of its precise age, Pando remains the largest known clonal organism, with a biomass of approximately 6,000 tonnes (as of 1996) [46]. Notably, a recently identified extensive female clone of the brown alga *Fucus vesiculosus* in the Baltic Sea may eventually surpass Pando in total biomass, pending further investigation [50].

These examples underscore the extraordinary longevity potential of plants at both the individual and clonal levels. Through diverse reproductive strategies, modular body plans, and resilience to environmental fluctuations, plants can persist for millennia—challenging traditional definitions of aging and offering unique insights into the biology of lifespan.

### Meristems as longevity safeguards

Multicellular organisms possess pluripotent stem cells to form new organs, replenish the daily loss of cells, or regenerate organs after injury. Stem cells are maintained in specific environments, the stem cell niches that provide signals to block differentiation. In plants, stem cell niches are situated in the shoot, root, and vascular meristems-self-perpetuating units of organ formation. Plants’ lifelong activity - which, as in the case of trees, can extend over more than a thousand years - requires that a robust regulatory network keeps the balance between pluripotent stem cells and differentiating descendants [51]. Totipotent regeneration abilities of plant stem cells made them an attractive target for biotechnology and agriculture applications, pioneered 80 years ago by micropropagation experiments of Ernest Ball [52, 53]. Even earlier, the unique and important character of plant meristem tissues was noticed: these tissues show an ability to avoid virus infection even in plants infected by viruses [54]. This feature has then been extensively exploited in agricultural practice for virus-free breeding. The molecular basis of this protection was demonstrated only recently to be based on RNA interference (RNAi) mechanism that actively degrades viral RNAs in shoot apical meristem (SAM) [55, 56]. The authors showed that a plant-encoded RNA-dependent RNA polymerase, a key player of the RNAi pathway, after activation by the plant hormone salicylic acid, amplifies antiviral RNAi in infected tissues. This provides stem cells with RNA-based virus sequence information, which prevents virus proliferation. Thus, not only a totipotency, but also the ability to resist virus infection and preserve its descendants (including clonally derived plants) from being infected, is a remarkable meristem feature.

**Fig. 2:**
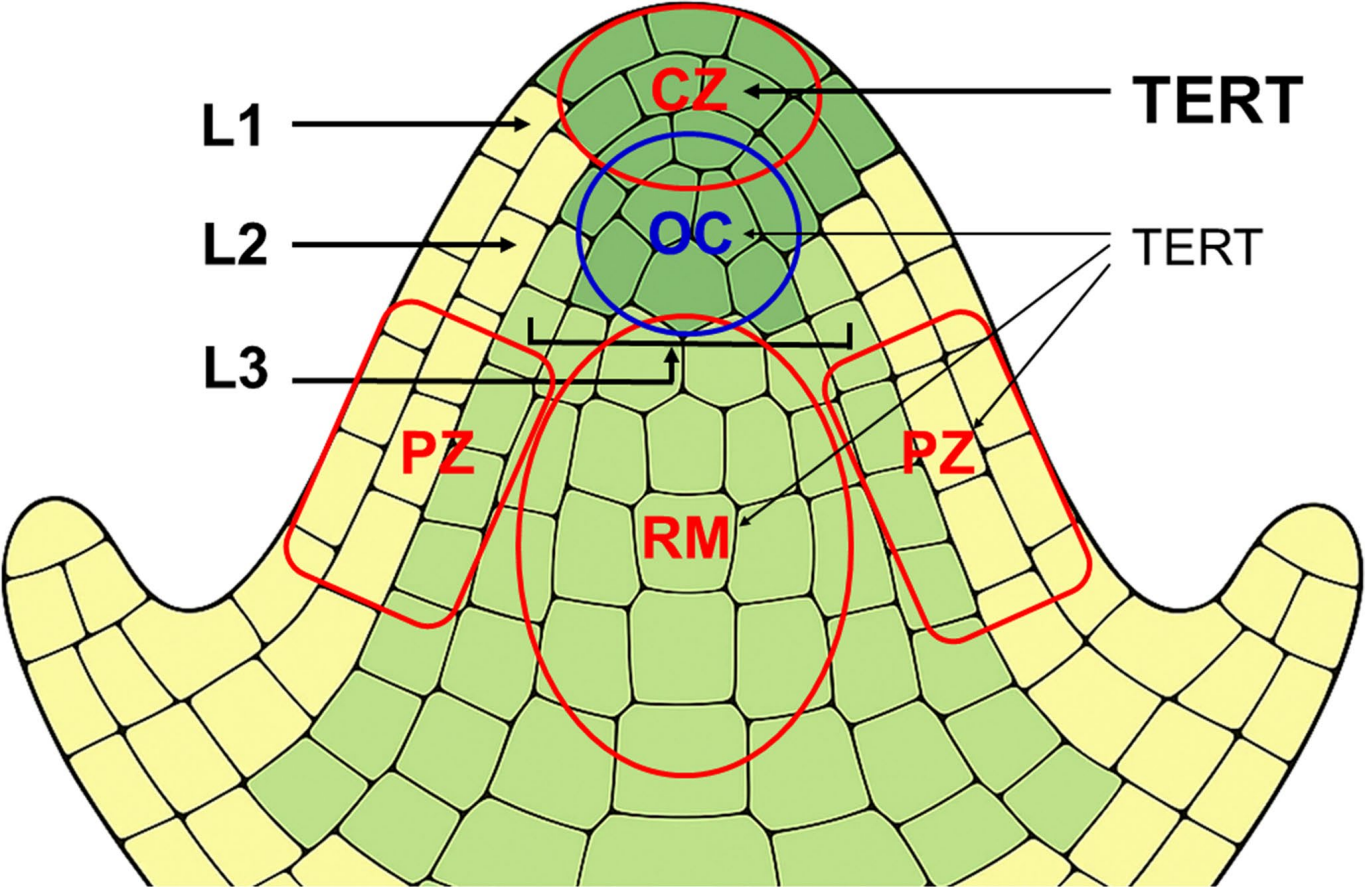
Schematic representation of the shoot apical meristem (SAM) located at the growing tip of the plant. Different functional zones are indicated. The central zone (**CZ**) contains slowly dividing stem cells, maintains stemness, and replenishes other SAM zones. The organizing centre (**OC**), located beneath and partially overlapping the CZ, emits signals (e.g. via *WUSCHEL*) that maintain stem cell identity in the CZ. The rib meristem (**RM**), positioned below the OC, produces cells that contribute to the stem and central tissues. The peripheral zone (**PZ**) surrounds the CZ and contains actively dividing, partially differentiated cells that give rise to lateral organs. Distinct cell layers—**L1**, **L2**, and the deeper **L3**—are marked with arrows. The two outermost layers, L1 and L2, are clonally distinct monolayers in which cells divide only anticlinally (i.e. perpendicular to the surface). In the deeper L3 layers, cells divide both anticlinally and periclinally (i.e. parallel to the surface). The labels “**TERT**” on the right indicate SAM regions with expression of the catalytic subunit of telomerase. Font sizes indicate differential expression levels (not to scale)

Stem cells, including plant stem cells, should be well **protected against accumulation of mutations**, one of the prominent aging factors. This can essentially be achieved either through an increased efficiency of DNA repair pathways, and/or a kind of protection against mutations originating namely during DNA replication. The increased capacity and fidelity of DNA repair were demonstrated in mammalian stem cells (e.g. [57], reviewed in [58]), as well as in plant meristem cells [59–61]. Moreover, the number of potentially dangerous replications, that may lead to the replicative senescence of the cells, is limited by the hierarchical, stratified organisation of meristems. E.g., SAM is organized into distinct layers (L1, L2, L3) with limited cell exchange between them (Fig. 2).

Consequently, mutations within specific layers, are prevented from widespread propagation throughout the plant. Studies have shown that the L2 layer of the shoot apical meristem, which contributes to the germline, accumulates mutations at a lower rate compared to the L1 layer. This suggests that plants may protect the germline by reducing mutation accumulation in the L2 layer [62]. The SAM is also organized into subdomains (zones) spanning across L1–L3. Stem cells are located in the central zone with directly beneath them the organization center, required to induce and maintain stem cell fate. Stem cells in the central zone divide very slowly, while their rapidly proliferating progeny cells are pushed laterally into the peripheral zone and further toward the primordia or basally into the organization centre or rib zone where they will adopt completely different cell fates [63]. Plant growth and tissue differentiation are driven by rapid divisions in the peripheral zone, whereas the central zone serves as a reservoir of cells with a low proliferation history that can repopulate the peripheral zone through occasional divisions [13].

An interesting concept of avoiding accumulation of mutations in stem cells is known as the “**immortal strand hypothesis**”, first proposed by John Cairns in 1975 [64]. He suggested that adult stem cells might minimize the accumulation of mutations by asymmetrically segregating their DNA strands during cell division, retaining the original (“immortal”) template strands in the self-renewing stem cell and passing the newly synthesized strands to the differentiating daughter cell. This mechanism was hypothesized to protect the stem cell genome from replication-induced errors. Since its proposal, the immortal strand hypothesis has been the subject of extensive research, with studies yielding both supporting and opposing evidence. Potten et al. (2002) [65] observed label-retaining cells in the small intestinal crypts of neonatal mice, suggesting selective retention of template DNA strands in stem cells. Conboy et al. (2007) [66] provided evidence of non-random template strand segregation in muscle stem cells during tissue regeneration, supporting the hypothesis. On the other hand, Tomasetti and Bozic (2015) [67] analyzed mutation accumulation in stem cells of various human tissue stem cells and found rates consistent with random DNA strand segregation, challenging the universality of the immortal strand mechanism. Also studies in developing zebrafish and other models have shown random segregation of DNA strands in stem cells, further questioning the general applicability of the hypothesis [68]. In this research, the authors employed metabolic labeling techniques using (2′S)−2′-deoxy-2′-fluoro-5-ethynyluridine (F-ara-EdU) and bromodeoxyuridine (BrdU), combined with Light sheet microscopy, to trace DNA strand inheritance during zebrafish development. Their observations revealed a rapid dilution of older DNA template strands in stem cell niches of the retina, brain, and intestine, indicating Random segregation of DNA strands during cell division. High-resolution microscopy of over 100 daughter cell pairs showed no evidence of asymmetric template strand segregation. In plants, experimental studies that would prove or disprove the immortal strand hypothesis, or that would possibly allow us to assess its universality, are still lacking. However, even if we rule out this hypothesis, plants employ numerous mechanisms mentioned above - such as reduced division rates, robust DNA repair, and spatial stem cell organization - that safeguard their meristem cells from mutation accumulation over long lifespans.

## Telomere maintenance and genome stability

Since the early 1970 s, the finite replicative lifespan of eukaryotic cells, termed the “Hayflick limit” [69]), has been attributed to the end-replication problem of linear chromosomes, as independently proposed by Olovnikov [70, 71] and Watson [72]. Chromosome termini—**telomeres**—have been widely regarded as intrinsic and potentially universal “aging clocks,” since their progressive shortening limits a cell’s replicative lifespan. Each cell division results in the loss of a portion of telomeric DNA repeats, eventually exposing chromosome ends, which activates DNA damage responses, induces cell cycle arrest, and leads to senescence [73–76]. This mechanism is highly conserved among eukaryotes and explains replicative limits observed in yeast, plants, and mammals.

However, **telomere shortening is not the sole determinant of organismal lifespan**. Early aging theories emphasized stochastic mutation accumulation [77] and antagonistic pleiotropy—where genes beneficial early in life exert deleterious effects later [78]. More recent models incorporate additional complexity, including **epigenetic drift** [79], **loss of proteostasis**,** mitochondrial dysfunction** [3], **chronic inflammation** (the “inflammaging” paradigm) [80], and **stem cell exhaustion** [81]. These hallmarks often interact: for example, reactive oxygen species (ROS) generated by mitochondria can damage telomeric DNA, accelerating its erosion, while epigenetic changes may alter the expression of telomerase or DNA repair genes. Thus, although telomere dynamics provide a measurable correlate of replicative aging, a **multifactorial framework** is needed to explain the diversity and plasticity of organismal lifespans.

Telomere shortening acts as a replicative “clock” only in cells lacking **telomere-maintenance mechanisms**. In most eukaryotes, this maintenance is performed by **telomerase**, a specialized ribonucleoprotein reverse transcriptase composed of a catalytic subunit (TERT) and an RNA template subunit (TR or TER) that directs the addition of telomeric repeats [82, 83]. In mammals, telomerase is active during early embryogenesis but is downregulated in most somatic tissues after the blastocyst stage—rendering these cells susceptible to telomere-driven replicative senescence. However, telomerase remains active in certain adult stem cell populations (e.g., germline, hematopoietic, intestinal crypt, and hair follicle stem cells) and is aberrantly reactivated in ~ 85% of cancers to enable immortalization [84, 85]. In approximately 10–15% of human cancers, telomere length is maintained via the **alternative lengthening of telomeres (ALT)** pathway. ALT utilizes homology-directed repair mechanisms such as recombination between telomeric repeats, rolling circle-replication of telomeric circles likely arising through these homologous recombination events, and break-induced replication. In mammalian cells, these processes occur in specialized nuclear foci called **ALT-associated PML bodies (APBs)** and are characterized by features such as telomere length heterogeneity, extrachromosomal C-circles, and elevated telomeric sister chromatid exchange (reviewed in [86, 87]).

Unfortunately, cancer cells can escape telomerase inhibition (e.g., by the drug imetelstat) by switching to ALT, continuing their proliferation. This underscores the rationale for **dual inhibition of telomerase and ALT** as a potentially more effective therapeutic strategy [88–90].

While ALT typically functions as a backup in telomerase-deficient contexts (e.g., in yeasts, worms, and mammals) [91–97]), it has evolved as the **primary telomere maintenance mechanism** in some taxa. For example, *Drosophila melanogaster* utilizes non-LTR retrotransposons (HeT-A, TART, and TAHRE) to extend telomeres [98, 99], and in chironomid midges (*Chironomus* spp.), telomere length is maintained by recombination among tandem satellite repeats [100].

Thus, telomere shortening reliably reflects cellular replicative history **only** when both telomerase and ALT pathways are inactive. The balance between telomere attrition and elongation ultimately governs telomere function as a mitotic lifespan regulator. Although telomere erosion is often linked to aging, **it is not a universal determinant of organismal longevity—even in humans**. Most individuals do not reach the so-called “telomeric brink” during their lifetime, suggesting that critically short telomeres are not the main trigger of aging-related decline [3, 101, 102]. These observations suggest that the relationship between telomere shortening, replicative senescence, and human longevity is more complex than a simple attainment of critically short telomeres [103].

### Telomere biology in plants: a different paradigm

Plant telomere dynamics diverge significantly from the animal model. Unlike mammals, where telomerase is developmentally silenced early and reactivated only in specialized stem cell niches [104], plants **maintain telomerase activity throughout life**, particularly in **meristematic tissues**. Furthermore, telomerase can be reactivated during regeneration or secondary meristem establishment from differentiated cells [105–107]. Consequently, classical models linking telomere shortening to aging **do not readily apply to plants**—or even universally to animals.

Interestingly, **ALT-like mechanisms** have also been documented in plants. For instance, *Arabidopsis* mutants lacking TERT [108] and *Physcomitrium* mutants lacking the TR subunit [95] show activation of recombination-based telomere maintenance. The unexpectedly **slow rate of telomere shortening** in *tert* mutants (250–500 bp per generation) and the rapid onset of ALT imply that **recombination-based telomere maintenance may operate even during normal development** [109].

Despite the absence of whole-organism aging in plants—facilitated by modular architecture and continuous meristem activity—organ- or cell-specific senescence, such as in leaves, still occurs. While these processes are hormonally regulated [110–112], their association with telomere shortening remains unclear. Crucially, **senescing cells do not divide**, precluding replicative telomere erosion. Instead, **telomeric DNA is degraded non-specifically**, as part of widespread chromatin breakdown under developmental or stress-induced conditions [113, 114]. Early misinterpretations of this degradation contributed to the false assumption that telomeres shorten developmentally during senescence [115].

### Does telomere length correlate with lifespan in plants?

The relationship between telomere length and plant lifespan is **non-linear and species-specific**. For example, the long-lived *Pinus longaeva* maintains stable, relatively short telomeres (6–11 kb) across individuals aged 54–3,500 years [116]. In contrast, the annual plant *Nicotiana tabacum* possesses telomeres up to 160 kb, with the shortest around 20 kb [117]. Research in short-lived annual species such as *Arabidopsis thaliana*, maize (*Zea mays*), and rice (*Oryza sativa*) indicates substantial natural variability in telomere length, without a clear positive correlation with longevity. Interestingly, longer telomeres in these species tend to correlate negatively with flowering time, reflecting developmental strategies rather than longevity per se [118]. Similarly, investigations into correlations between genome or chromosome sizes and telomere lengths in plants have shown inconsistent or absent relationships. Specifically, *A. thaliana* ecotypes (of similar genome sizes) exhibit no significant correlation between genome size and telomere length [118]. Telomeres range from 2 to 9 kb, with variations observed among different ecotypes and even among individual plants within the same population [119]. *Silene latifolia* and *N. tabacum*—plants with comparable genome sizes—show contrasting telomere lengths of 2.5–4.5 kb and 20–160 kb, respectively [107]. These observations underscore the **complexity of telomere regulation in plants** and invalidate the assumption that telomere length universally predicts lifespan or chromosome size.

### It is not the length of telomeres per se that matters, but telomere homeostasis

Telomere length homeostasis in dividing cells depends on a balance between **positive and negative regulatory elements**. Positive regulators include primarily telomerase and factors that facilitate telomerase assembly and recruitment [120], whereas negative regulators—primarily telomere-binding proteins—restrict telomerase access and modulate telomeric chromatin structure [121]. In yeast, a “protein-counting” model posits that telomere shortening reduces available binding sites for telomere proteins acting as repressors (e.g., Rap1p), thereby permitting telomerase-mediated extension until a repressive threshold is restored [122]. A similar competitive model has been proposed in human cells, where limited telomerase availability results in preferential elongation of the shortest telomeres [120]. While these models effectively explain **average telomere length regulation**, they do not account for the **consistent variation in length among different telomeres within a single cell**. Recent advances in genomic technologies—particularly **nanopore-based telomere profiling**—have enabled the high-resolution mapping of **chromosome arm-specific telomere lengths**, revealing that each telomere maintains a unique and stable length equilibrium.

#### Telomerase: a central player in plant telomere homeostasis

In plants, **the reversible regulation of telomerase activity**, particularly in association with cell proliferation and tissue regeneration, has attracted increasing interest. Unlike in animals, telomerase remains **active in meristematic tissues** throughout plant life and can be rapidly reactivated in differentiated cells that resume division. This developmental plasticity eliminates replicative telomere shortening during plant ontogenesis and shifts research focus toward understanding the molecular components that regulate this system [123, 124]. The core components of telomerase, TERT and TR have been primary targets of these studies. Identification of TERT was relatively simple due to its conserved domain structure among eukaryotes. As in other systems, its expression correlates with telomerase activity [125, 126]. On the other hand, identification of plant TRs has been challenging due to their generally poor sequence conservation and was achieved only recently [127]. Interestingly, while TR expression is **constitutive in animals** [128], **TR transcript levels are upregulated in proliferating plant tissues** [127]. This is somewhat surprising, given that plant TR genes—like those in all Archaeplastida—are transcribed by RNA polymerase III and contain a **type-3 promoter** composed of a TATA box and upstream sequence element (USE) [95], reviewed in [129]. Therefore, current research is focused on elucidateing the composition and structure of the plant telomerase ribonucleoprotein complex, in particular TR-interacting proteins beyond TERT, epitranscriptomic modifications of TR and the other factors that may be important to control TR stability in plant cells. Progress in understanding the **structure and folding dynamics of TR**, as well as its interaction with TERT during telomerase biogenesis [130, 131] may provide critical insights into the **reversible regulation of telomerase** in plants. Identifying novel TR-TERT interactors and fully characterizing the plant telomerase holoenzyme remain key goals in this field.

#### Telomere-binding proteins in telomere function and homeostasis

In parallel to telomerase, protein components of telomere chromatin have been identified and functionally characterized in plants. Among these, **Telomere Repeat-Binding (TRB)** proteins—also referred to as **Single MYB Histone (Smh)** family proteins—are of particular importance. TRBs are unique to plants and exhibit a **tripartite domain structure** comprising an N-terminal SANT/myb-like domain (for sequence-specific DNA binding), a central H1-like globular domain (associated with non-specific DNA binding and protein–protein interactions), and a C-terminal coiled-coil domain [132]. TRBs bind telomeric DNA both in vitro and in vivo via their MYB-like domain, while the H1-like domain mediates critical protein interactions [133–136]. These include **TRB homo- and heterodimerization**, interaction with telomere-associated proteins such as **AtPOT1b**, a *PROTECTION OF TELOMERES 1* paralog in *Arabidopsis* [137, 138], and interaction with **RuvBL1 and RuvBL2a**, plant homologs of the human telomerase-associated proteins Pontin and Reptin [139]. Although in humans Pontin and Reptin directly interact with TERT, in *Arabidopsis*, this interaction appears to be **indirect**, likely mediated by TRBs—a hypothesis supported by co-localization studies and telomere shortening observed in *trb1* mutants. These findings suggest that **TRBs contribute to telomerase recruitment** and may participate in broader telomere regulatory networks [140]. Notably, TRBs also possess **epigenetic regulatory functions**, which are discussed in the last chapter.

Another important telomere-associated complex in plants is the **CST complex**, a conserved heterotrimer consisting of **CTC1**,** STN1**,** and TEN1** subunits [141]. CST is integral to telomere maintenance and genome stability across eukaryotes, including plants, and facilitates the replication of telomeric DNA. It coordinates **C-strand synthesis** by recruiting **DNA polymerase α-primase** following G-strand extension by telomerase [142]. CST also limits telomerase access by binding to **single-stranded telomeric DNA**, thus **preventing over-elongation** and contributing to length homeostasis. Beyond telomeres, CST helps resolve **replication stress** by stabilizing stalled forks and thereby maintaining genome integrity [143]. In *Arabidopsis*, CST interacts with telomerase and telomere-associated proteins such as **POT1a**, and mutations in CST components result in **telomere dysfunction**,** developmental abnormalities**, and **reduced viability**, underscoring its critical role in plant development [144].

### Toward chromosome-specific regulation of telomere length

Although species-, tissue-, or cell-type-specific telomere length regulation can be explained by the interplay of telomerase, binding proteins, and associated factors, these models do **not** explain why **telomeres on different chromosome arms within the same cell** can maintain distinct, yet stable, lengths.

This phenomenon points to an additional layer of **cis-acting**,** chromosome-specific regulation**. Recent advancements in **nanopore sequencing of telomere-enriched samples** have enabled **Telomere Profiling** at near-nucleotide resolution, revealing that each human telomere possesses a unique length equilibrium [145]. While species-specific telomere length is controlled by a cellular balance of positive and negative regulators, **the specific setting of individual telomere lengths remains unexplained**.

A recent **yeast study** provides compelling support for a **cis-acting regulatory mechanism**. Artificially increasing **Sir4 protein** abundance at a specific subtelomeric region resulted in the selective lengthening of that telomere, without affecting others [146]. Sir4, in yeast, serves both as a silencing factor for subtelomeric genes and as a recruiter of telomerase via its interaction with **KU80**, which binds telomerase RNA. While the telomere position effect and KU80–TR interaction have not been demonstrated in plants [147], the core principle of **epigenetic**,** telomere-specific length regulation** may still apply across species [148]. In plants, such regulation could involve distinct chromatin states [117, 149, 150] or unknown **plant-specific factors** that mediate analogous control mechanisms. The discovery and characterization of these elements represent a promising direction for future research.

## Epigenetic scripts in leaf senescence

Plant telomeres serve not only as structural end-caps that protect chromosome termini but also as **integrative components of the epigenetic regulatory network**. Functional interactions between telomeres, telomere-binding proteins, and chromatin regulators—such as the Polycomb Repressive Complex 2 (PRC2) and the PEAT complex—suggest that telomeres participate in broader networks controlling plant development, stress responses, and aging.

This chapter builds on that perspective, exploring how epigenetic mechanisms—including histone modifications, DNA methylation, and chromatin remodeling—shape the transcriptional landscape during senescence and contribute to the chromatin-level programming of plant aging.

### Epigenetic regulation of gene expression in senescence

Epigenetic regulation, which modulates gene expression without altering the underlying DNA sequence, plays a pivotal role in developmental transitions such as the shift from growth to senescence. **Dynamic and reversible changes in chromatin structure** integrate developmental cues and environmental signals, influencing **transcriptional programs** that affect plant stress responses, development, and lifespan.

Recent studies, primarily in the model plant *Arabidopsis thaliana* and increasingly in crop species, have begun to uncover how epigenetic changes contribute to plant senescence. These findings also reveal **parallels and distinctions in senescence and aging between plants and animals** (Fig. 1). This chapter explores the current understanding of how epigenetic mechanisms regulate leaf senescence, highlighting their roles in coordinating internal and external signals during the aging trajectory.

In both *Arabidopsis* and crop plants, evidence shows that epigenetic changes underlie the senescence process. Transcriptomic analyses indicate that approximately 16% of *Arabidopsis* genes exhibit **altered expression during senescence-related reprogramming** [151, 152]. This includes the upregulation of senescence-associated genes (SAGs) and the repression of senescence-downregulated genes (SDGs). Key transcription factors such as ORESARA1 (ORE1), NAP, and WRKY53 activate SAGs involved in nutrient mobilization and chlorophyll degradation (e.g., *NON-YELLOW COLORING 1 (NYC1)*, *NON-YELLOWING 1/2 (NYE1/2)* [23]). In contrast, SDGs—primarily related to photosynthesis and biosynthetic pathways—are repressed, reflecting functional decline in aging tissues [153].

Notably, many SAGs also play roles in stress responses. For example, *SAG13* influences seed germination under oxidative stress and protects against pathogens [154, 155]. Similarly, drought stress-responsive genes such as *RESPONSIVE TO DEHYDRATATION 20* (*RD20*) and *RELATED TO AP2.4* (*RAP*2.4) are also upregulated during senescence [151].

### DNA methylation dynamics during senescence

DNA methylation regulates gene expression by modifying chromatin accessibility. In plants, cytosine methylation occurs in symmetrical (CG, CHG) and asymmetrical (CHH) contexts (H = A, T, or C). Different DNA methyltransferases accomplish methylation in specific sequence contexts: DNA METHYLTRANSFERASE 1 (MET 1) maintains CG methylated, CHROMOMETYLASE 3 (CMT3) has the same role in CHG, and DOMAINS-REARRANGED METHYLTRANFERASEs (DRMs) *de novo* methylate cytosines in all contexts [156]. Inverse process, DNA demethylation, is catalyzed by REPRESSOR OF SILENCING 1 (ROS1), DEMETER (DME), and DEMETER-LIKE (DML) proteins [157]. **DNA methylation**, mainly CG methylation, typically **silences genes through modification of promoters** (transcriptional gene silencing) **or gene bodies** (posttranscriptional gene silencing). Repetitive sequences, including transposable elements (TEs), are methylated mainly in CHH motives, which prevents their mobilization [158].

Genetic studies in *A. thaliana* demonstrate the role of DNA methylation in senescence. Reduced MET1 activity exhibits global DNA hypomethylation and pleiotropic developmental effects manifested in plenty of phenotypic and developmental traits, including delayed senescence onset [159] whereas *ros1* and *drm1/2 cmt3* mutants show accelerated senescence [160]. Pilot studies reported **a global reduction in DNA methylation during senescence**, with downregulation of *MET1* and *CMT3* and upregulation of *ROS1*, *DME*, and *DML* [161]. However, subsequent studies showed **more nuanced patterns**: for example, natural senescence in *Arabidopsis* is associated with a mild decrease in CG methylation and an increase in CHH methylation [160]. Interestingly, dark-induced senescence did not significantly alter global methylation levels, though some hypomethylated CHH regions were detected [162]. Transcriptomic analysis during leaf senescence showed stable or reduced expression of DNA methylation-related genes, including *MET1* and *CMT2/3* [163], contrasting with previous findings [161].

Reports from other species are inconsistent. Increased DNA methylation during senescence has been found in *Pinus radiata* (radiata pine), *Prunus persica* (peach), *Phyllostachys heterocycla* (moso bamboo), *Pinus tabuliformis* (Chinese pine), and *Eucalyptus urophylla*×*Eucalyptus grandis* hybrid [164–169]. Conversely, species like *Acacia mangium* (hickory wattle), chestnut, and *Sequoiadendron giganteum* (giant redwood) exhibit decreased cytosine methylation during senescence [170–172].

**Senescence** is often accompanied by **TEs reactivation**, but the **correlation with DNA methylation loss is unclear**, either linked to demethylation [173], or occurring independently [162]. Nevertheless, the methylation-independent transcriptional activation of Arabidopsis TEs was also observed in other studies, which were not related to plant senescence [174, 175].

**In humans and other vertebrates** (e.g., mice, dogs, wolves), **age-associated DNA methylation changes occur consistently among individuals**, and the combined methylation status of selected CGs correlates with the chronological age. Recently, differences between DNA methylation age and true chronological age are even supposed to reflect biological age (reviewed in [176]). Inconsistencies reported for the relationship between DNA methylation and plant senescence thus suggest that **DNA methylation changes during plant senescence** are highly **species-specific and context-dependent**. Methodological differences, such as genome-wide vs. site-specific analyses, may also explain discrepancies. Future high-resolution methylome studies at the single-plant level may clarify whether methylation patterns represent universal features of senescence or reflect lineage-specific programs.

### Histone modifications and the histone code

Histone modifications—especially on histone **H3 N-terminal tails**—are central to chromatin structure and gene regulation. Generally, marks as H3K9 acetylation and H3K4 di- or tri-methylations are associated with euchromatic regions encompassing actively transcribed genes. In contrast, H3K9me2 marks plant constitutive heterochromatin, and H3K27 di- and tri-methylations are linked to transcriptionally inactive genes [177]. Loading and erasing of these histone marks are dynamically regulated by histone acetyltransferases and methyltransferases, and histone deacetylases and demethylases, respectively [178, 179]. Specific combinations of histone marks form a “**histone code**” that determines chromatin structure and transcriptional output.

**Fig. 3:**
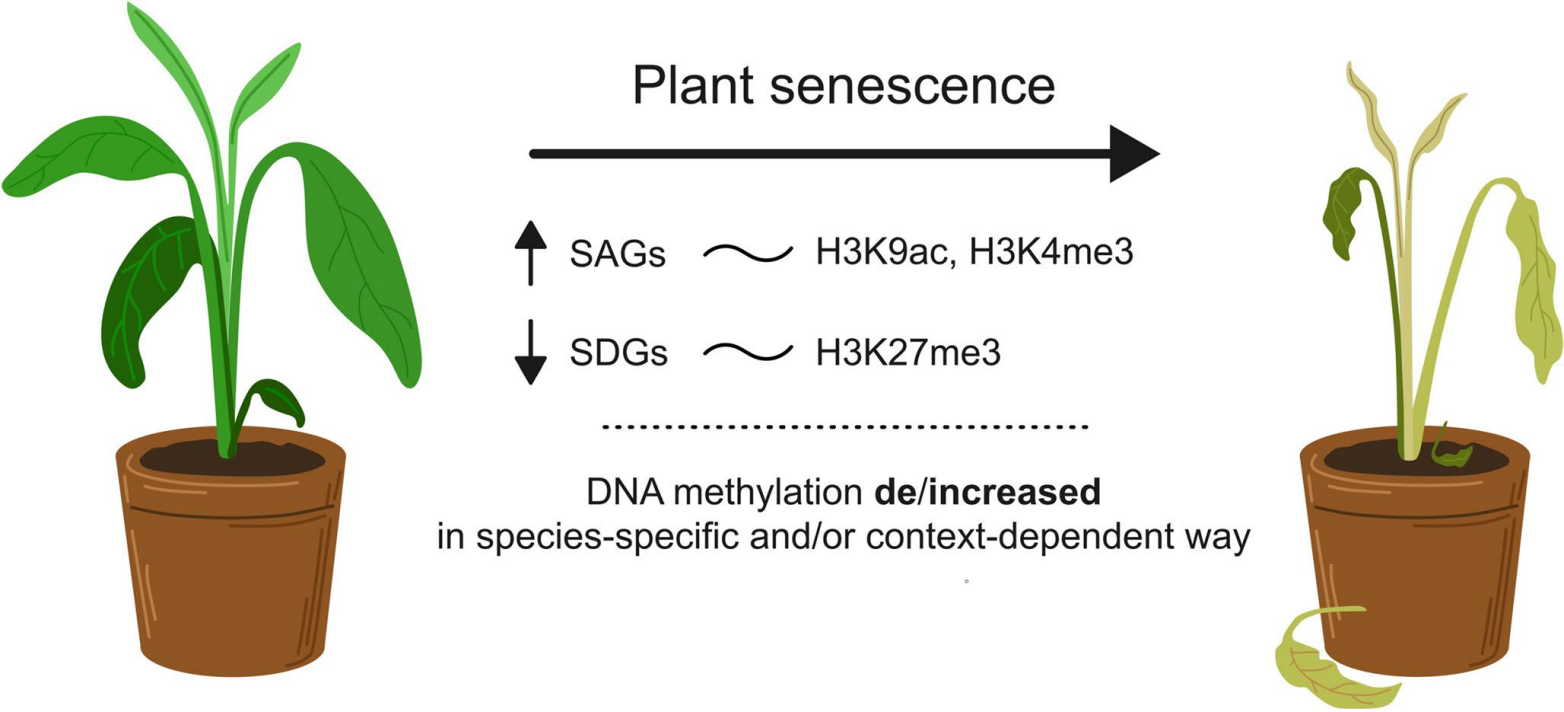
Epigenetic regulation of gene expression during plant leaf senescence. Plant leaf senescence is driven by multiple layers of epigenetic mechanisms that modify chromatin structure and gene activity. DNA methylation dynamics, including context-dependent methylation and demethylation, contribute to changes in transcription, although these patterns vary among species and may depend on the specific methodological approach. Histone modifications such as H3K9 acetylation and H3K4 trimethylation activate senescence-associated genes (SAGs), while H3K27 trimethylation suppresses genes downregulated during senescence (SDGs). The interaction of these epigenetic layers enables plants to integrate developmental signals and environmental cues, thereby regulating the progression of senescence

The impact of histone modifications and enzymes responsible for their maintenance on the onset of leaf senescence has been broadly studied and summarized (e.g. [163, 180–182]). Notably, the combinatorial pattern of histone modifications may play the key role in global structural changes of chromatin during senescence, as many of these events are not correlated with changes of cytosine methylation (see above). During leaf senescence (Fig. 3), SAGs are typically associated with increased H3K9ac and H3K4me3, while SDGs are repressed through H3K27me3 accumulation [183, 184]. For instance, *WRKY53* activation correlates with elevated H3K4me2/3 in its regulatory regions, and overexpression of respective histone demethylase delays senescence [185]. Similarly, histone acetyltransferases and deacetylases act antagonistically to regulate senescence timing [180, 186, 187]. These findings underscore the critical role of histone modifications in transcriptional reprogramming during senescence. Unlike DNA methylation, histone modifications often act more directly and reversibly, **allowing fine-tuned control of gene expression during aging**.

### Chromatin remodeling and structural dynamics

Chromatin remodelers—ATP-dependent complexes that **reposite**,** evict or restructure nucleosomes**—are essential for broad chromatin reorganization during senescence. Mutants in remodelers such as DRD1 and DDM1, which also interface with DNA methylation pathways [188, 189]—show delayed senescence and impaired SAG activation [190]. Overexpression of ORESARA7 (ORE7), an A-T hook protein, also delays senescence, likely by limiting chromatin relaxation and transcription factor access to SAG promoters [191]. These remodelers **not only regulate local gene accessibility** but also o**rchestrate global chromatin transitions** necessary for the **progression of senescence**.

### Intersections of telomere and epigenetic regulation

Epigenetic regulations of genes and telomeres are not associated only through common general mechanisms but in at least some cases, also through the same players. In addition to terminal telomere DNA arrays, *A. thaliana* TRB1 binds promoters of ribosome biogenesis genes, modulating their expression positively or negatively [192]. **TRB1 functions as a bivalent transcriptional modulator**, maintaining Polycomb Group (PcG) target gene repression in LIKE HETEROCHROMATIN PROTEIN1 (LHP1) deficient mutants, while promoting expression of PcG-independent targets [193]. H3K27me3 deposition at telomeres and centromeres—regulated by PRC2 and influenced by histone H1—is significantly increased in *h1* mutants [194]. **TRB4 and TRB5 interact with PRC2 components** like SWINGER and CURLY LEAF, affecting the expression of flowering genes such as *FT* and *SOC1* [123, 124]. TRB proteins also form part of the PEAT complex (PWO, EPCR, ARID, TRB), which is involved in chromatin remodeling and gene regulation, and TRBs play a role of their targeting subunit [195, 196]. Additionally, TRBs associate with the histone demethylase JUMONJI14 (JMJ14) which removes H3K4me3 from target genes. *trb1/2/3* and *jmj14-1* mutants show elevated H3K4me3 and transcriptional activation of these genes [197]. Collectively, these results highlight **TRBs as multifaceted regulators that bridge telomere biology and broader epigenetic control of development and senescence.** Importantly, the telomeric and non-telomeric (epigenetic) functions of TRB proteins cannot be fully understood when considered separately.

Thus, epigenetic regulation is fundamental to leaf senescence, coordinating gene expression in response to developmental and environmental signals. While DNA methylation patterns vary across species, histone modifications and chromatin remodeling consistently emerge as major regulators of chromatin reconfiguration during senescence. Importantly, telomere-binding proteins like TRBs link telomere maintenance with broader chromatin regulation, suggesting an integrated epigenetic control network. Understanding these complex regulatory layers holds promise for modulating plant aging and improving crop resilience.

## Conclusions

Plant aging and senescence represent highly dynamic and multifactorial processes shaped by the modular, totipotent, and regenerative nature of plant growth. While telomere dynamics have often been emphasized in aging theories, accumulating evidence suggests that in plants, telomere length alone does not serve as a universal aging clock. Instead, epigenetic regulation—through DNA methylation, histone modifications, and chromatin remodeling—plays a central role in orchestrating developmentally programmed senescence and stress responses. By integrating chromosomal and chromatin-level mechanisms, we gain a more nuanced understanding of how plants maintain longevity and resilience. These insights not only refine our models of plant aging but also offer promising avenues for improving crop performance and sustainability in the face of environmental challenges.

## Data Availability

Data sharing is not applicable to this article as no new data were created or analyzed in this study.
